# Prophylactic mRNA Vaccination against Allergy Confers Long-Term Memory Responses and Persistent Protection in Mice

**DOI:** 10.1155/2015/797421

**Published:** 2015-10-18

**Authors:** E. Hattinger, S. Scheiblhofer, E. Roesler, T. Thalhamer, J. Thalhamer, R. Weiss

**Affiliations:** Department of Molecular Biology, University of Salzburg, 5020 Salzburg, Austria

## Abstract

Recently, mRNA vaccines have been introduced as a safety-optimized alternative to plasmid DNA-based vaccines for protection against allergy. However, it remained unclear whether the short persistence of this vaccine type would limit memory responses and whether the protective immune response type would be maintained during recurrent exposure to allergen. We tested the duration of protective memory responses in mice vaccinated with mRNA encoding the grass pollen allergen Phl p 5 by challenging them with recombinant allergen, 3.5, 6, and 9 months after vaccination. In a second experiment, vaccinated mice were repeatedly challenged monthly with aerosolized allergen over a period of 7 months. Antibody and cytokine responses as well as lung inflammation and airway hyperresponsiveness were assessed. mRNA vaccination induced robust TH1 memory responses for at least 9 months. Vaccination efficiently suppressed TH2 cytokines, IgE responses, and lung eosinophilia. Protection was maintained after repeated exposure to aerosolized allergen and no TH1 associated pathology was observed. Lung function remained improved compared to nonvaccinated controls. Our data clearly indicate that mRNA vaccination against Phl p 5 induces robust, long-lived memory responses, which can be recalled by allergen exposure without side effects. mRNA vaccines fulfill the requirements for safe prophylactic vaccination without the need for booster immunizations.

## 1. Introduction

Due to a constant rise in incidence of type I allergic diseases the need for effective treatment options is apparent. However, specific immunotherapy (SIT), the only treatment currently available, is time-consuming and entails many disadvantages such as the potential to create new sensitizations and serious side effects, including anaphylaxis. Moreover, the inevitable transition from extract-based SIT to component-resolved diagnosis and therapy of allergic diseases with recombinant molecules seems to be a lengthy process. An alternative concept to SIT with recombinant molecules includes DNA immunization with allergen genes, an approach which meanwhile has entered the clinical study phase [1–3].

In the past years, the urgent need to fight the worldwide increasing incidence of allergies also drew attention to “true vaccination” against allergic diseases, that is, prophylactic immunization of healthy individuals [4, 5]. The identification of children at high risk to develop allergy has improved significantly [6, 7], thus facilitating the selection of target groups for prophylactic interventions. However, allergen extracts licensed for treatment of established allergies will not be applicable for prophylactic immunization due to safety issues and the risk to induce* de novo* sensitizations [8–10]. Only modified (hypoallergenic) allergen derivatives and gene vaccines can be considered as suitable candidates for prophylactic allergy vaccines. Among gene vaccines, mRNA conforms best to the stringent requirements for vaccines against type I allergy. Due to its short* in vivo* persistence mRNA acts in an “immunize and disappear” way, thus limiting expression of encoded allergens [11]. Furthermore, and in contrast to DNA vaccines, mRNA vaccines lack control sequences and cannot integrate into the host genome. These properties led to the classification of non-replicative mRNA as non-gene therapy by regulatory authorities [12]. Application of mRNA has so far proven its effectiveness for vaccination against infectious diseases and tumors in animal models [13, 14] and also in clinical studies with mRNA encoding tumor-associated antigens [15, 16]. With regard to type I allergies we have demonstrated that mRNA vaccines induce a protective TH1-type response against a panel of different allergens, leading to inhibition of specific IgE production and prevention of lung inflammation and airway hyperresponsiveness (AHR) in mice [17]. Despite the proof that mRNA vaccines are effective and protect against allergic sensitization in murine models, doubts about their long-term efficacy remained. There have been concerns that short-term antigen expression might result in weak memory responses unable to protect from future encounters [18].

Therefore, in the present paper, one set of experiments investigates the long-term protection after mRNA vaccination (up to nine months after vaccination). A second approach deals with the robustness of the protective response. The immune system of patients under real-life conditions is exposed to allergen repetitively over weeks and months, or even perennial, depending on the allergen. This is in contrast to typical experimental setups which usually perform a few allergen challenges within a short time period. Hence we simulated the human situation of seasonal pollen exposure by a repeated challenge of vaccinated mice with aerosolized grass pollen allergen (up to seven months after vaccination).

## 2. Materials and Methods

### 2.1. Preparation of mRNA Vaccines

The plasmid encoding Phl p 5, pTNT-P5, has been described [17]. Plasmids for RNA transcription were purified using an EndoFree Plasmid Giga Kit (Qiagen, Düsseldorf, Germany). For RNA transcription, plasmids were linearized and templates were purified via phenol-chloroform-isoamyl alcohol extraction, followed by a single chloroform-isoamyl alcohol extraction. Plasmids were precipitated by adding a 1/10 volume of 3 M sodium acetate (pH 5.2) and two volumes of 100% ethanol on ice and washed three times with 70% ethanol. All transcription reactions were performed with T7 or SP6 RiboMAX Large Scale RNA Production Systems (Promega, Mannheim, Germany). Residual template DNA was removed by means of digestion with RNAse-free DNAse (Promega, Mannheim, Germany). After transcription, the RNA was precipitated by ammonium acetate precipitation (addition of 1 volume 5 M ammonium acetate, 15 min on ice) followed by centrifugation, washed with 70% ethanol, and resuspended in nuclease-free H_2_O. Capping was performed* in vitro* by using a ScriptCap m^7^G Capping Kit (Epicentre Biotechnologies, Madison, USA), following the manufacturer's instructions.

### 2.2. Animals and Immunizations

BALB/c mice, aged between 6 and 14 weeks, were obtained from Charles River Laboratories (Sulzfeld, Germany) and were maintained according to the local guidelines for animal care. All animal experiments were approved by the Austrian Ministry of Science.

To evaluate the duration of the protective effect of mRNA immunization, 5 mice per group were immunized intradermally (i.d.) three times in one-week intervals with 100 *μ*g of capped-Phl p 5 mRNA. Non-vaccinated mice served as a control group. 3.5, 6, or 9 months after vaccination the animals were sensitized by two subcutaneous (s.c.) injections of 200 *μ*L PBS containing 1 *μ*g recombinant Phl p 5 (Biomay, Vienna, Austria) and 100 *μ*L Alu-Gel-S (1.3% suspension with an aluminium content of 5.9–7.1 mg/mL, Serva Electrophoresis GmbH, Heidelberg, Germany) in a 10-day interval. Seven days after the second sensitization, mice were exposed to nebulized recombinant Phl p 5 in PBS for 30 min on three consecutive days. Therefore, 5 mL recombinant Phl p 5 in PBS (0.2 mg/mL) was nebulized using a PARI BOY SX nebulizer with a PARI LL nebulizer head (PARI, Starnberg, Germany) in a 25 × 25 × 25 cm nebulization chamber.

To analyze the effect of repeated allergen challenge, 5 animals per group were vaccinated i.d. three times in one-week intervals, with either 100 *μ*g of capped-Phl p 5 or capped-Bet v 1 control (mock) mRNA (encoding the irrelevant allergen Bet v 1). Non-vaccinated mice served as a control group. All groups were sensitized twice by s.c. injection of 200 *μ*L PBS containing 1 *μ*g rPhl p 5 and 100 *μ*L Alu-Gel-S 33 and 42 days after the last vaccination. Seven days after the second sensitization, the mice were challenged three times with 1 mg nebulized rPhl p 5 in PBS. On the next day, Penh was measured to assess AHR. These challenges were repeated monthly over a period of seven months. Sera were collected at regular intervals during the course of the experiments.

### 2.3. Serology: ELISA and Mediator Release Assay

**Figure 1:**
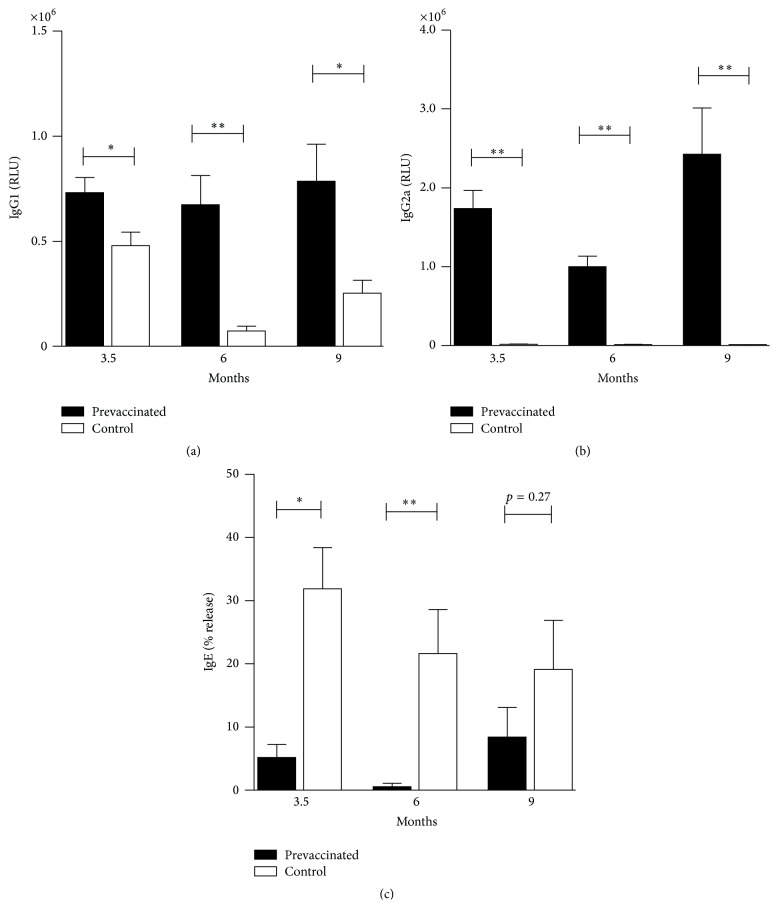
Phl p 5-specific IgG1 (a), IgG2a (b), and IgE (c) antibodies one week after sensitization. BALB/c mice were prevaccinated or left untreated and sensitized with recombinant Phl p 5 after the indicated time interval. Data are shown as relative light units of a luminometric ELISA or as percentage of total release induced by addition of 10% Triton X-100 and presented as means ± SEM (*n* = 5). ^*∗*^ *p* < 0.05; ^*∗∗*^ *p* < 0.01.

Antigen-specific IgG1 and IgG2a antibody levels in sera were determined by using a luminescence-based ELISA, as described [19]. Sera were diluted 1 : 100,000 for IgG1 and 1 : 10,000 for IgG2a determination. Functional IgE levels were assessed by using a rat basophil leukemia (RBL) cell release assay as described previously [20]. For the determination of IgE levels, sera were diluted 1 : 100 (Figure 1) or 1 : 150 (Figure 4).

### 2.4. Lymphocyte Cultures and Cytokine Detection

To determine cytokine secretion by splenocytes, spleens were isolated and single cell suspensions were prepared as described [19]. Cells were restimulated* in vitro* in the absence or presence of 10 *μ*g/mL recombinant Phl p 5 for 48 h. Cytokine expression in the culture supernatants was analyzed with a FlowCytomix Kit (eBioscience, Schwechat, Austria), following the manufacturer's instructions.

### 2.5. Bronchoalveolar Lavage

Bronchoalveolar lavage (BAL) was performed as described [21]. In short, cytokines were determined by FlowCytomix assay and cells were stained for FACS analysis with the following markers: anti-CD19-PE/Cy7, anti-CD45-PerCP/Cy5.5, anti-CD4-APC/Cy7, anti-Gr1-APC (all BioLegend, London, UK), anti-CD8-FITC (eBioscience, Schwechat, Austria), and anti-CD25-PE (BD Biosciences, Schwechat, Austria). Red blood cells were lysed and cells were analyzed on a FACSCanto II flow cytometer (BD Biosciences, San Jose, CA). The eosinophil population was distinguished by CD45^med^Gr1^low^side-scatter^high^ phenotype. Neutrophils were identified as a CD45^high^, Gr1^high^ cell population.

### 2.6. Whole-Body Plethysmography

To measure the overall airway obstruction, non-invasive unrestrained whole-body plethysmography (WBP) was performed using a Buxo WBP system consisting of a Bias Flow Regulator, 6 WBP chambers, a MAX II preamplifier unit, and BioSystem XA Software (Buxco, Winchester, UK). The animals were put into individual chambers and exposed to nebulized 0.9% NaCl followed by increasing concentrations of nebulized methacholine (5 mg/mL; 10 mg/mL dissolved in 0.9% NaCl) followed by 0.9% NaCl for a recovery phase. 50 *μ*L of methacholine or NaCl per chamber was applied to the nebulizer head and enhanced pause in breathing (Penh) was measured for 5 min at each concentration. Data were analyzed as the area under the curve [22].

### 2.7. Dynamic Lung Resistance and Compliance Measurement

Resistance and dynamic compliance were measured with a FinePointe Series RC site (Buxco, Winchester, UK), according to the manufacturer's instructions. Mice were anesthetized by means of intraperitoneal ketamine/xylazine injection, and the trachea was surgically exposed, cannulated, and connected to the ventilator. Transpulmonary pressure was measured by inserting an esophageal cannula. Baseline signals for resistance and dynamic compliance were recorded, and mice were exposed to aerosolized PBS containing increasing amounts of methacholine (5 mg/mL; 10 mg/mL). Values for each dose are expressed as raw values or percentage of baseline values.

### 2.8. Statistical Analysis

**Figure 2:**
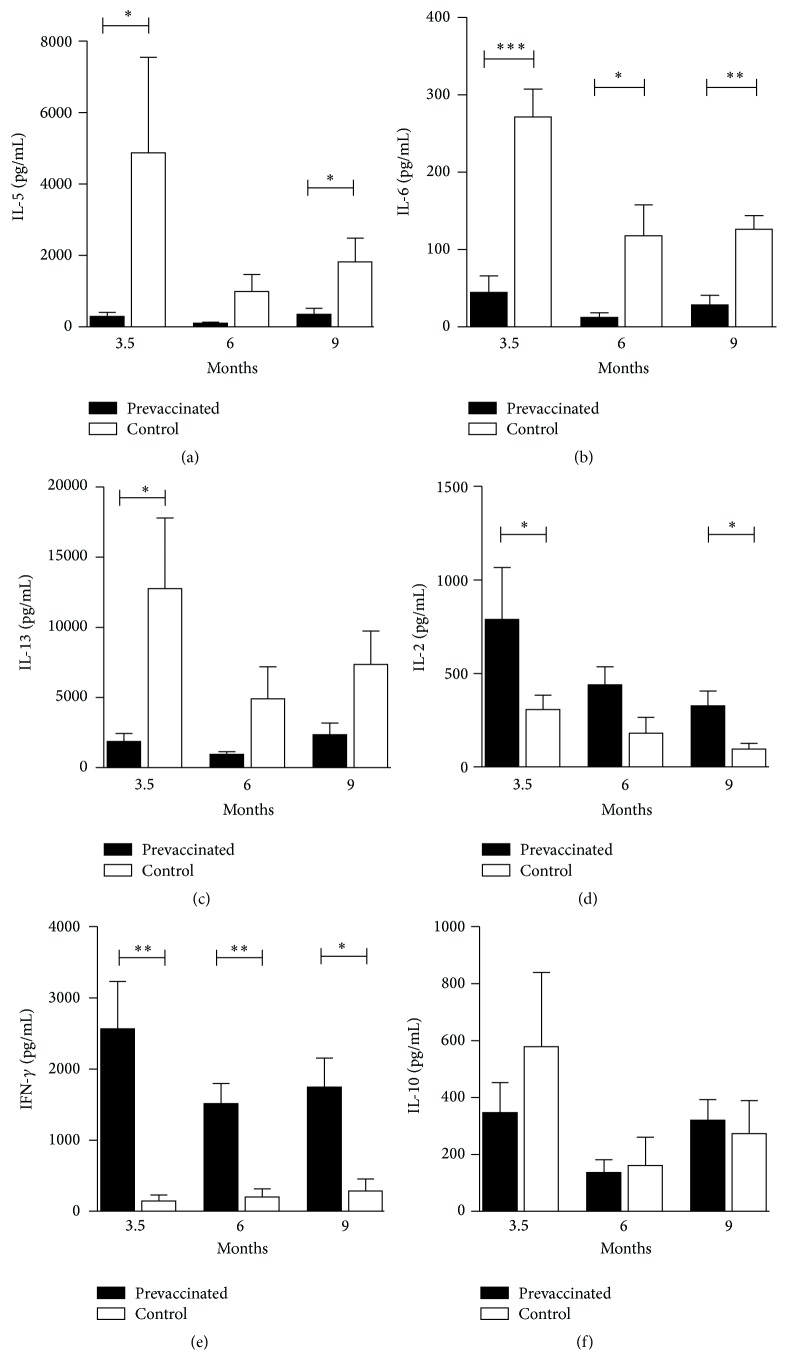
Levels of IL-5 (a), IL-6 (b), IL-13 (c), IL-2 (d), IFN-*γ* (e), and IL-10 (f) were determined in culture supernatants after* in vitro* restimulation of splenocytes with rPhl p 5. Data are displayed as means ± SEM (*n* = 5). ^*∗*^ *p* < 0.05; ^*∗∗*^ *p* < 0.01; ^*∗∗∗*^ *p* < 0.001.

**Figure 3:**
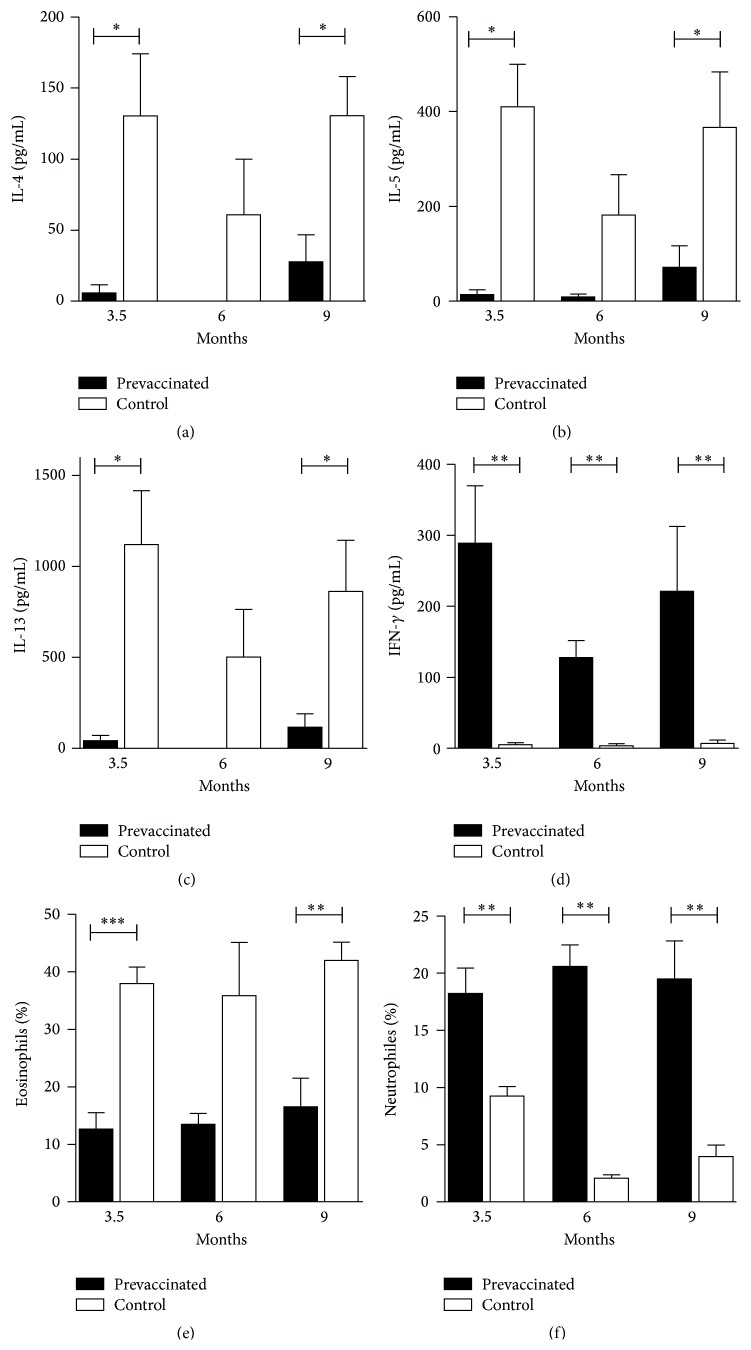
Levels of IL-4 (a), IL-5 (b), IL-13 (c), and IFN-*γ* (d) as well as the percentage of eosinophils (e) and neutrophils (f) of total leukocytes in BAL fluids were assessed. Data are shown as means ± SEM (*n* = 5). ^*∗*^ *p* < 0.05; ^*∗∗*^ *p* < 0.01; ^*∗∗∗*^ *p* < 0.001.

Differences between means of vaccinated versus control sample groups were analyzed by unpaired *t*-test (Figures 1–3). Comparisons between multiple groups (Figures 5 and 6) were done by one-way ANOVA followed by Tukey's post hoc test. All statistical analyses were performed using GraphPad Prism 5.

## 3. Results

### 3.1. RNA Vaccination Induces Long-Term Protective Memory Responses

To evaluate the long-term memory and duration of the protective effect after mRNA immunization, animals were vaccinated three times in one-week intervals with 100 *μ*g of capped-Phl p 5 mRNA i.d. and sensitized with recombinant allergen after 3.5, 6, and 9 months, respectively. Sensitization was performed by s.c. injection of 1 *μ*g recombinant Phl p 5 in alum. Seven days later, mice received three challenges with nebulized recombinant Phl p 5, on three consecutive days, in order to induce TH2-mediated lung inflammation. The effect of mRNA vaccination on the humoral immune response was determined by measuring IgG1 and IgG2a levels after the last aerosol challenge. We found antigen-specific IgG1 significantly elevated in prevaccinated mice compared to control animals even 9 months after the initial vaccination (Figure 1(a)), suggesting the presence of B cell memory. More strikingly, only prevaccinated mice showed Phl p 5-specific IgG2a, indicating the maintenance of an RNA vaccine-induced TH1 memory for at least 9 months (Figure 1(b)). Levels of functional Phl p 5-specific IgE were assessed by RBL release assay and data shows that mRNA vaccination reduced allergen-specific IgE levels at all three time points (Figure 1(c)). This data clearly demonstrates that RNA vaccination induces a long-lasting humoral immune response and maintains a TH1-biased memory, which prevents IgE induction for at least 6 months following prophylactic immunization.

To evaluate the long-term effects of mRNA vaccination on T cell responses, splenocytes of prevaccinated and control animals were harvested at each time point and cytokine secretion upon restimulation with antigen was measured. In contrast to cells from prevaccinated mice, splenocytes from control animals displayed elevated secretion of TH2 cytokines, including IL-5, IL-6, and IL-13 (Figures 2(a)–2(c)). In return, prevaccination significantly increased the expression of the TH1-type cytokines IL-2 and IFN-*γ* even 9 months after vaccination, compared to cells from control mice (Figures 2(d)-2(e)). This data confirms that RNA vaccine-induced TH1 memory responses are robust and long-lasting. No significant differences in IL-10 expression could be detected between prevaccinated and control groups (Figure 2(f)). IL-17 production was below the detection limit, whereas levels of IL-21 and IL-22 were detectable but not influenced by vaccination (data not shown).

Exposure to inhalant allergens, such as the grass pollen allergen Phl p 5, can cause the emergence of inflammation in the lung and the development of asthma. To test the efficacy of mRNA vaccination to protect from lung inflammation, BAL fluids were analyzed. The levels of IFN-*γ* were found to be significantly higher in the BAL fluids of prevaccinated mice (Figure 3(d)) and correlated with reduced levels of TH2-type cytokines. BAL fluids of prevaccinated mice contained less IL-4, IL-5, and IL-13 (Figures 3(a)–3(c)) compared to control groups. Eosinophil recruitment, one of the hallmarks of allergic lung inflammation, could be measured in all groups after the challenge with aerosolized allergen but was significantly reduced in prevaccinated mice (Figure 3(e)), whereas the percentage of infiltrating neutrophils in the lung was increased in the vaccinated groups (Figure 3(f)).

An important characteristic of allergic asthma is increased AHR. We assessed the effects of mRNA vaccination on AHR by measuring resistance and dynamic compliance in response to increasing concentrations of aerosolized methacholine. No statistically significant differences between prevaccinated and control mice could be detected, concerning neither resistance nor compliance of the lungs. However, by trend, vaccinated groups showed reduced resistance and increased compliance. This also indicates that the increase in neutrophils during the acute phase had no detrimental effect on lung function (Supplementary Figure S1 in Supplementary Material available online at http://dx.doi.org/10.1155/2015/797421).

### 3.2. RNA Vaccination Maintains Long-Term Protection over Repeated Aerosol Challenges

To determine the robustness of the protective immune response and whether repeated aerosol exposure would either abrogate the TH1-mediated protective effect by reconversion to an allergic TH-2 response or induce TH1-driven side effects, BALB/c mice were prevaccinated with an mRNA vaccine encoding Phl p 5 or the irrelevant antigen Bet v 1 (mock control) and sensitized with recombinant Phl p 5 one month later. Subsequently, mice were challenged monthly by exposure to aerosolized recombinant Phl p 5 on three consecutive days over a period of seven months. IgG1 and IgG2a levels were determined by ELISA after each challenge with aerosolized allergen.

**Figure 4:**
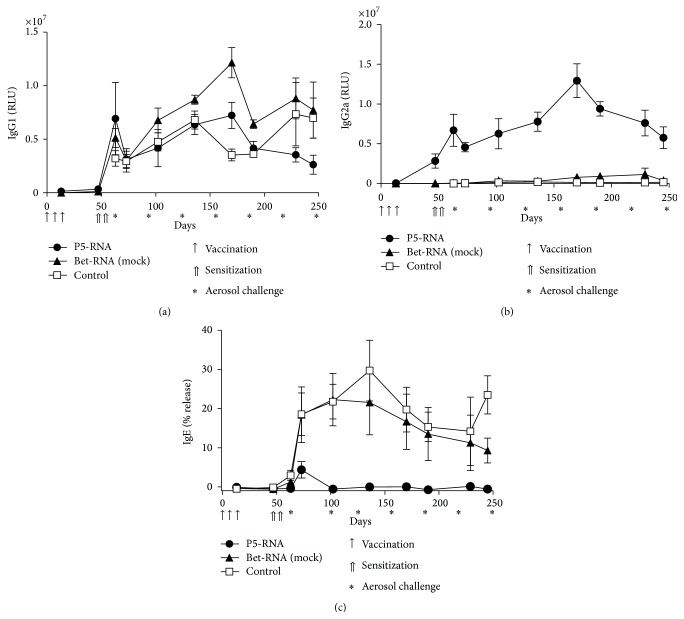
Time course of Phl p 5-specific IgG1 (a), IgG2a (b), and IgE (c) antibody levels as measured by a luminometric ELISA or RBL assay, respectively. BALB/c mice were prevaccinated and sensitized one month later, followed by monthly exposure to aerosolized rPhl p 5. Data are displayed as means ± SEM (*n* = 5).

**Figure 5:**
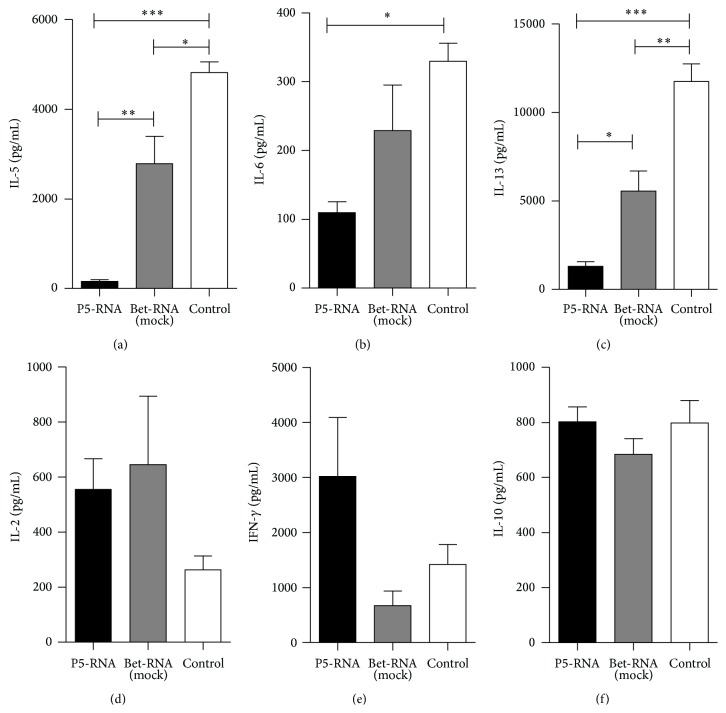
Levels of IL-5 (a), IL-6 (b), IL-13 (c), IL-2 (d), IFN-*γ* (e), and IL-10 (f) in supernatants of* in vitro* restimulated splenocyte cultures. Cells were obtained from mice immunized 9 months before, sensitized, and repeatedly exposed to aerosolized rPhl p 5. Data are displayed as means ± SEM (*n* = 5). ^*∗*^ *p* < 0.05; ^*∗∗*^ *p* < 0.01; ^*∗∗∗*^ *p* < 0.001.

Elevated levels of antigen-specific IgG2a were present in sera from prevaccinated mice throughout the experiment, indicating the maintenance of TH1-type memory induced by mRNA vaccination (Figure 4(b)). Prevaccinated mice also showed higher Phl p 5-specific IgG2a titers compared to the mock-RNA control groups, clearly pointing to the specificity of the mRNA vaccination. Phl p 5-specific IgG1 antibodies did not differ between the groups (Figure 4(a)). Importantly, vaccination with mRNA strongly suppressed the induction of Phl p 5-specific IgE antibodies. Levels of IgE were significantly reduced in the prevaccinated group even after mice had been challenged 21 times with aerosolized recombinant Phl p 5 over a period of seven months (Figure 4(c)). This correlated with significantly reduced secretion of TH2 cytokines IL-5, IL-6, and IL-13 by splenocytes from prevaccinated mice, which had been restimulated* in vitro* (Figures 5(a)–5(c)). In contrast, IL-2 and IFN-*γ* were increased in prevaccinated mice (Figures 5(d)-5(e)), indicating the maintenance of a TH1-biased response. We further confirmed that vaccination does not induce Tr1 cells, as shown by similar levels of IL-10 secretion in all groups (Figure 5(f)).

**Figure 6:**
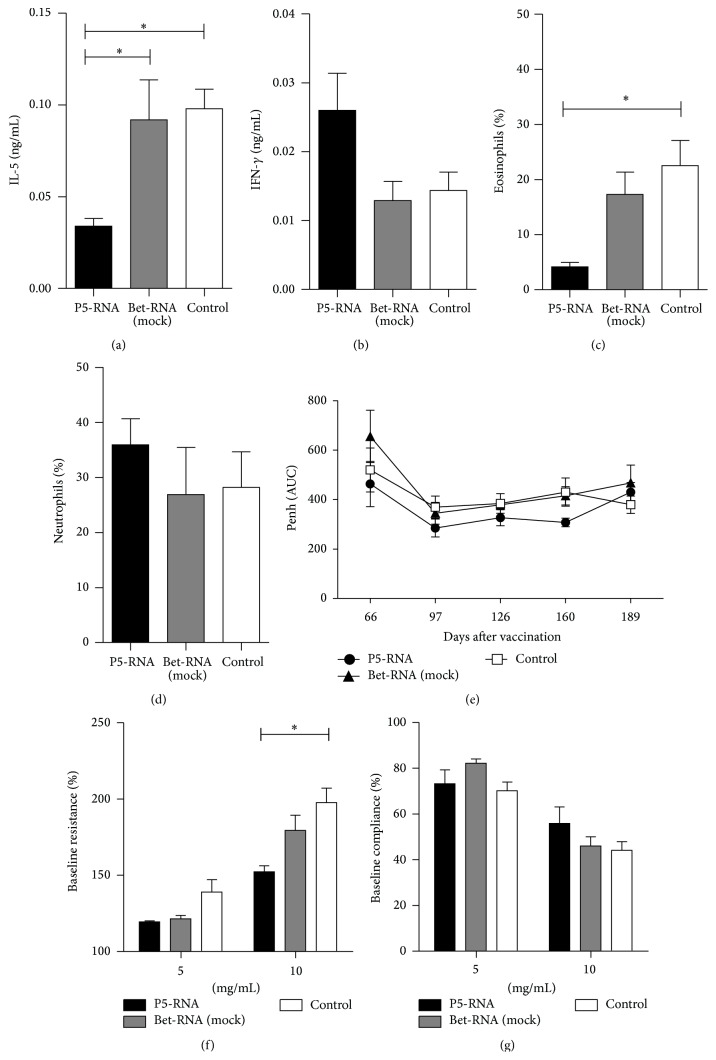
Levels of IL-5 (a) and IFN-*γ* (b) as well as the percentage of eosinophils (c) and neutrophils (d) of total leukocytes in BAL fluids determined 9 months after mRNA immunization. Effects of prevaccination on lung function were determined by measuring airway hyperresponsiveness via noninvasive whole-body plethysmography throughout the experiment (e) or invasive measurement of lung resistance (f) and dynamic compliance (g) at the endpoint. Mice were exposed to nebulized 0.9% NaCl followed by increasing concentrations of nebulized methacholine. Data are shown as means ± SEM (*n* = 5). ^*∗*^ *p* < 0.05.

In addition to the systemic immune response, we also investigated the response in the target organ of the aerosol challenge, the lung. Similar to the cytokine expression by* in vitro* stimulated splenocytes, levels of IFN-*γ* were higher in BAL fluids from prevaccinated mice (Figure 6(b)), and this IFN-*γ* induction correlated with reduced IL-5 secretion, as prevaccinated mice had significantly decreased IL-5 levels in BAL fluids compared to control groups (Figure 6(a)). Furthermore, antigen specificity of protection was confirmed as levels of IFN-*γ* and IL-5 expression in mock-RNA vaccinated groups were similar to the control group (Figures 6(a) and 6(b)). As seen in the acute phase response (Figure 3), the percentage of eosinophils infiltrating into the lung was significantly reduced in prevaccinated mice despite repeated aerosol challenges (Figure 6(c)). The percentage of neutrophils in the lung infiltrate was relatively high. However, this increased infiltration of neutrophils was not dependent on prevaccination and was present in all groups (Figure 6(d)).

To assess AHR during the course of this experiment we measured enhanced pause (Penh) with noninvasive whole-body plethysmography. Mice pretreated with the mRNA vaccine overall showed lower AHR compared to mock-RNA treated or control animals (Figure 6(e)). Additionally, after the final airway challenge, AHR was directly assessed using invasive R/C measurement. In agreement with the immunological results, these data also indicate a protective effect of the prevaccination with mRNA, which improved lung function compared to the nonvaccinated group (Figures 6(f) and 6(g)).

## 4. Discussion

We have previously demonstrated that mRNA vaccination can protect against a broad range of allergens in a mouse model of allergic asthma [17]. However, concerns have been raised that the short persistence of mRNA vaccines might induce insufficient memory responses. In the current study, we show for the first time that the protective, antiallergic TH1 memory is long-lasting and sufficient to prevent allergic sensitization up to 9 months after the initial vaccination. Sensitization, that is, induction of a TH2-biased immune response, is inhibited with respect to both branches of the immune system. Vaccination with mRNA encoding the grass pollen allergen prevents expression of TH2 cytokines, such as IL-5 and IL-13, and proinflammatory IL-6 by* in vitro* restimulated splenocytes, thus reflecting the systemic effect. It also inhibits the induction of a TH2-mediated lung inflammation as indicated by reduction of TH2 cytokines in the lung. IFN-*γ* in BAL fluid was elevated in vaccinated mice, especially in the acute phase of the response. IFN-*γ* plays an ambiguous role in allergic lung inflammation. On the one hand, it directly inhibits TH2 cells, induces apoptosis in eosinophils, reduces levels of lung IgE and airway hyperresponsiveness, and directly acts on lung epithelial cells, thus blocking mucus production [23–25]. On the other hand, chronic expression of IFN-*γ* has also been shown to enhance allergen induced eosinophilia, IL-5, and IL-13 expression [24] and has induced side effects when administered to allergic patients [25]. These data illustrate that IFN-*γ* has potent immunomodulatory capacities that can be highly beneficial but also induce side effects at high dosages. In our model, no detrimental effects were observed.

The induction of regulatory T cells has been shown to play an important role in keeping or restoring a nonallergic balanced status of the immune system against allergens [26]. However, in our model no significant differences in IL-10 expression could be detected between prevaccinated and control groups indicating no crucial role of IL-10-secreting Treg cells (Tr1) in the mechanisms underlying protection from an allergic immune response by mRNA immunization (Figure 2(f)).

Furthermore, recruitment of eosinophils to the lung was significantly reduced in the vaccination groups. Interestingly, the percentage of infiltrating neutrophils in the lung was increased in the vaccinated group during the acute phase of the lung response (Figure 3(f)). Similarly, Duechs et al. observed that application of various TLR agonists in an asthma model reduced airway eosinophilia and airway resistance but at the same time increased neutrophil influx [27]. It has been shown that IL-17 derived from TH17 cells [28] or iNKT cells [29] can induce recruitment of neutrophils to the lung. TH17 cells have also recently been found to be involved in the pathology of allergy [30]. However, in our model IL-17 production was below the detection limit, whereas levels of IL-21 and IL-22 were detectable but no alterations could be seen in the vaccination groups (data not shown). IFN-*γ* has also been shown to boost trafficking of neutrophils into the lung [31], and the elevated levels of this cytokine during the acute phase in the vaccinated groups may therefore contribute to the enhanced influx of neutrophils. During the chronic phase of inflammation, due to repeated exposure to aerosolized allergen, the percentage of neutrophils in the lung infiltrate was relatively high. However, this increased infiltration of neutrophils was not dependent on prevaccination but was present in all groups (Figure 6(d)). TNF-*α*, a cytokine secreted by TH1 cells, may also be responsible for recruitment of eosinophils to the lung [32]. In the acute phase of the response we found low levels of TNF-*α* in splenocytes which were higher in vaccinated mice compared to control animals, at least at the earlier challenge time points (Supplementary Figure S2A). TNF-*α* levels were higher after chronic allergen exposure in all groups (Supplementary Figure S2B). Neutrophil invasion therefore most likely represents a general side effect of chronic exposure to allergen and is only induced by mRNA vaccination during the acute sensitization phase. Nevertheless, it must be emphasized that even in the acute phase this vaccine-induced influx of neutrophils does not impair lung function, as AHR is not increased in these groups.

Our data further ascertained that mRNA vaccination induces a robust protection and inhibits induction of a TH2-type response even after repeated exposure to a high dose of allergen. Thus, repeated allergen exposure during the pollen season does neither lead to mitigation of the established TH1-biased response nor does it induce TH1-driven lung pathology. In contrast, it acts like booster immunizations and thus resembles the mechanisms by which lifelong specific immunity can be maintained against certain pathogens, after a single vaccination. Likewise one or two injections of an mRNA vaccine would be sufficient to trigger the allergen-specific recruitment of protective TH1 memory cells and, in the case of seasonal allergens, the natural exposure acts as boost and refreshment of the protective response type. Moreover, prophylactic mRNA vaccination against allergens does not need as strong immune responses as classical vaccination approaches against pathogens or tumors. An almost nondetectable primary immune response induced by the mRNA vaccine is sufficient to set an immunological bias, which prevents subsequent sensitization against the allergen [17, 21, 33].

## 5. Conclusions

mRNA vaccination prevents an allergen-specific TH2-type response by suppressing TH2 cytokines, eosinophils, and IgE expression, while increasing TH1-type parameters such as IFN-*γ* expression. Collectively, our data indicate that mRNA vaccines are effective in inducing a protective, robust, and long-lasting TH1-biased immune response.

mRNA vaccines therefore combine effective prevention of allergic sensitization with a commendable safety profile.

## Supplementary Material

Supplementary Figure 1 shows lung resistance and dynamic compliance results of mice sensitized 3.5, 6, or 9 months after vaccination.
Supplementary Figure S2 provides TNF-[alpha] levels in supernatants of splenocytes restimulated with rPhl p 5 in the acute and the chronic sensitization model.

## Abbreviations

AHR: Airway hyperresponsiveness
BAL: Bronchoalveolar lavage
SIT: Specific immunotherapy
Penh: Enhanced pause.

## References

[1] Weiner L., Mackler B., Hearl B., Fitz-Patrick D. (2014). Poster 2011: a DNA vaccine immunotherapy for japanese red cedar allergy. *World Allergy Organization Journal*.

[2] NCT01707069 A Safety and Immunogenicity Phase I Study of CryJ2-DNA-Lysosomal Associated Membrane Protein (CryJ2 -DNA-LAMP) Plasmid. NCT01707069.

[3] Immunomic Therapeutics http://www.immunomix.com/japanese-red-cedar.

[4] Valenta R., Campana R., Marth K., van Hage M. (2012). Allergen-specific immunotherapy: from therapeutic vaccines to prophylactic approaches. *Journal of Internal Medicine*.

[5] Holt P. G., Sly P. D., Sampson H. A. (2013). Prophylactic use of sublingual allergen immunotherapy in high-risk children: a pilot study. *Journal of Allergy and Clinical Immunology*.

[6] Kulig M., Bergmann R., Niggemann B., Burow G., Wahn U. (1998). Prediction of sensitization to inhalant allergens in childhood: evaluating family history, atopic dermatitis and sensitization to food allergens. *Clinical and Experimental Allergy*.

[7] Campbell D. E., Boyle R. J., Thornton C. A., Prescott S. L. (2015). Mechanisms of allergic disease—environmental and genetic determinants for the development of allergy. *Clinical & Experimental Allergy*.

[8] Ball T., Sperr W. R., Valent P. (1999). Induction of antibody responses to new B cell epitopes indicates vaccination character of allergen immunotherapy. *European Journal of Immunology*.

[9] Van Ree R., Van Leeuwen W. A., Dieges P. H. (1997). Measurement of IgE antibodies against purified grass pollen allergens (Lol p 1, 2, 3 and 5) during immunotherapy. *Clinical & Experimental Allergy*.

[10] Movérare R., Elfman L., Vesterinen E., Metso T., Haahtela T. (2002). Development of new IgE specificities to allergenic components in birch pollen extract during specific immunotherapy studied with immunoblotting and Pharmacia CAP System. *Allergy*.

[11] Weiss R., Scheiblhofer S., Roesler E., Weinberger E., Thalhamer J. (2012). MRNA vaccination as a safe approach for specific protection from type i allergy. *Expert Review of Vaccines*.

[12] Weide B., Garbe C., Rammensee H.-G., Pascolo S. (2008). Plasmid DNA- and messenger RNA-based anti-cancer vaccination. *Immunology Letters*.

[13] Fotin-Mleczek M., Duchardt K. M., Lorenz C. (2011). Messenger RNA-based vaccines with dual activity induce balanced TLR-7 dependent adaptive immune responses and provide antitumor activity. *Journal of Immunotherapy*.

[14] Petsch B., Schnee M., Vogel A. B. (2012). Protective efficacy of *in vitro* synthesized, specific mRNA vaccines against influenza A virus infection. *Nature Biotechnology*.

[15] Kubler H., Stenzl A., Schultze-Seemann W. (2011). Final analysis of a phase I/IIa study with CV9103, an intradermally administered prostate cancer immunotherapy based on self adjuvanted mRNA. *European Journal of Cancer*.

[16] Sebastian M., von Boehmer L., Zippelius A. (2012). Messenger RNA vaccination and B-cell responses in NSCLC patients. *Journal of Clinical Oncology*.

[17] Roesler E., Weiss R., Weinberger E. E. (2009). Immunize and disappear-safety-optimized mRNA vaccination with a panel of 29 allergens. *Journal of Allergy and Clinical Immunology*.

[18] Gray D., Matzinger P. (1991). T cell memory is short-lived in the absence of antigen. *Journal of Experimental Medicine*.

[19] Hartl A., Weiss R., Hochreiter R., Scheiblhofer S., Thalhamer J. (2004). DNA vaccines for allergy treatment. *Methods*.

[20] Hochreiter R., Stepanoska T., Ferreira F. (2003). Prevention of allergen-specific IgE production and suppression of an established Th2-type response by immunization with DNA encoding hypoallergenic allergen derivatives of Bet* *v* *1, the major birch-pollen allergen. *European Journal of Immunology*.

[21] Gabler M., Scheiblhofer S., Kern K. (2006). Immunization with a low-dose replicon DNA vaccine encoding Phl p 5 effectively prevents allergic sensitization. *Journal of Allergy and Clinical Immunology*.

[22] Hamelmann E., Schwarze J., Takeda K. (1997). Noninvasive measurement of airway responsiveness in allergic mice using barometric plethysmography. *American Journal of Respiratory and Critical Care Medicine*.

[23] Mitchell C., Provost K., Niu N., Homer R., Cohn L. (2011). IFN-gamma acts on the airway epithelium to inhibit local and systemic pathology in allergic airway disease. *Journal of Immunology*.

[24] Koch M., Witzenrath M., Reuter C. (2006). Role of local pulmonary IFN-*γ* expression in murine allergic airway inflammation. *American Journal of Respiratory Cell and Molecular Biology*.

[25] Teixeira L. K., Fonseca B. P. F., Barboza B. A., Viola J. P. B. (2005). The role of interferon-*γ* on immune and allergic responses. *Memorias do Instituto Oswaldo Cruz*.

[26] Akdis C. A., Akdis M. (2014). Mechanisms of immune tolerance to allergens: role of IL-10 and Tregs. *The Journal of Clinical Investigation*.

[27] Duechs M. J., Hahn C., Benediktus E. (2011). TLR agonist mediated suppression of allergic responses is associated with increased innate inflammation in the airways. *Pulmonary Pharmacology and Therapeutics*.

[28] Ye P., Rodriguez F. H., Kanaly S. (2001). Requirement of interleukin 17 receptor signaling for lung CXC chemokine and granulocyte colony-stimulating factor expression, neutrophil recruitment, and host defense. *Journal of Experimental Medicine*.

[29] Michel M.-L., Keller A. C., Paget C. (2007). Identification of an IL-17–producing NK1.1^neg^ iNKT cell population involved in airway neutrophilia. *Journal of Experimental Medicine*.

[30] Liu Y., Zeng M., Liu Z. (2015). Clinical relevance of Th17 response in allergic rhinitis: more evidence. *Clinical & Experimental Allergy*.

[31] McLoughlin R. M., Witowski J., Robson R. L. (2003). Interplay between IFN-*γ* and IL-6 signaling governs neutrophil trafficking and apoptosis during acute inflammation. *The Journal of Clinical Investigation*.

[32] Lukacs N. W., Strieter R. M., Chensue S. W., Widmer M., Kunkel S. L. (1995). TNF-*α* mediates recruitment of neutrophils and eosinophils during airway inflammation. *Journal of Immunology*.

[33] Pulsawat P., Pitakpolrat P., Prompetchara E. (2013). Optimization of a Der p 2-based prophylactic DNA vaccine against house dust mite allergy. *Immunology Letters*.

