# Supplementary material for: Prophylactic mRNA Vaccination against Allergy Confers Long-Term Memory Responses and Persistent Protection in Mice
Source: J Immunol Res. 2015 Oct 18;2015:797421. doi: 10.1155/2015/797421 (PMC4628660; doi:10.1155/2015/797421)
Supplement: Supplementary file 1 — Supplementary Figure 1 shows lung resistance and dynamic compliance results of mice sensitized 3.5, 6, or 9 months after vaccination. Supplementary Figure S2 provides TNF-[alpha] levels in supernatants of splenocytes restimulated with rPhl p 5 in the acute and the chronic sensitization model. [file 797421.f1.docx]

## Prophylactic mRNA vaccination against allergy confers long-term memory responses and persistent protection in mice

E. Hattinger, S. Scheiblhofer, E. Roesler, T. Thalhamer, J. Thalhamer & R. Weiss

Department of Molecular Biology, University of Salzburg, Salzburg, Austria

Correspondence: Richard Weiss, Department of Molecular Biology, University of Salzburg, Hellbrunnerstrasse 34, 5020 Salzburg, Austria; Tel +4366280445737; E-mail: [Richard.Weiss@sbg.ac.at](mailto:Richard.Weiss@sbg.ac.at)

## Online Supplement


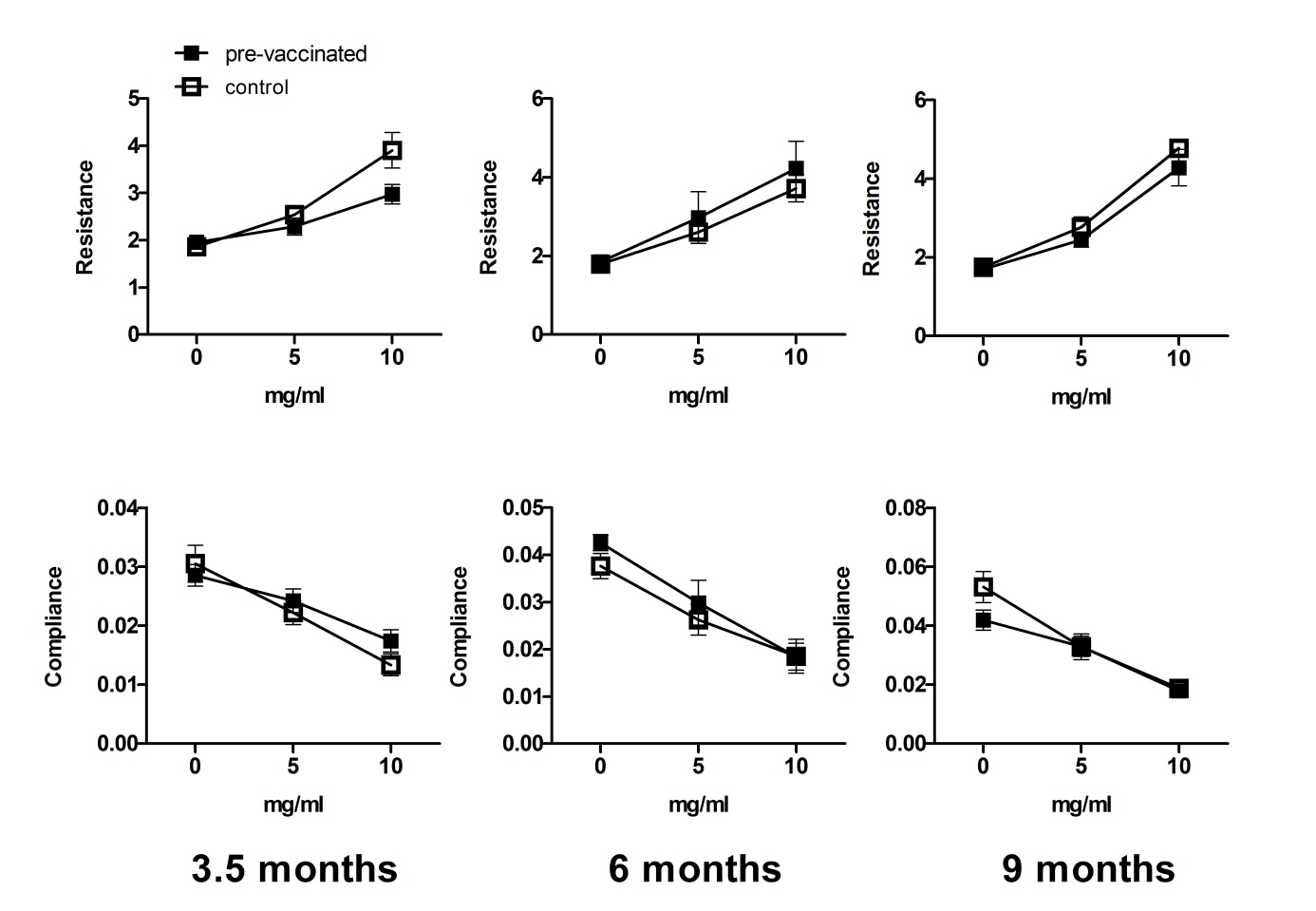


Supplementary Figure S1. Mice were vaccinated with mRNA, sensitized, and challenged once after the indicated time interval. Effects of pre-vaccination on lung function were tested by measuring lung resistance (top panels) and dynamic compliance (bottom panels) in response to increasing concentrations of methacholine. Data are shown as mean ± SEM (n = 5).


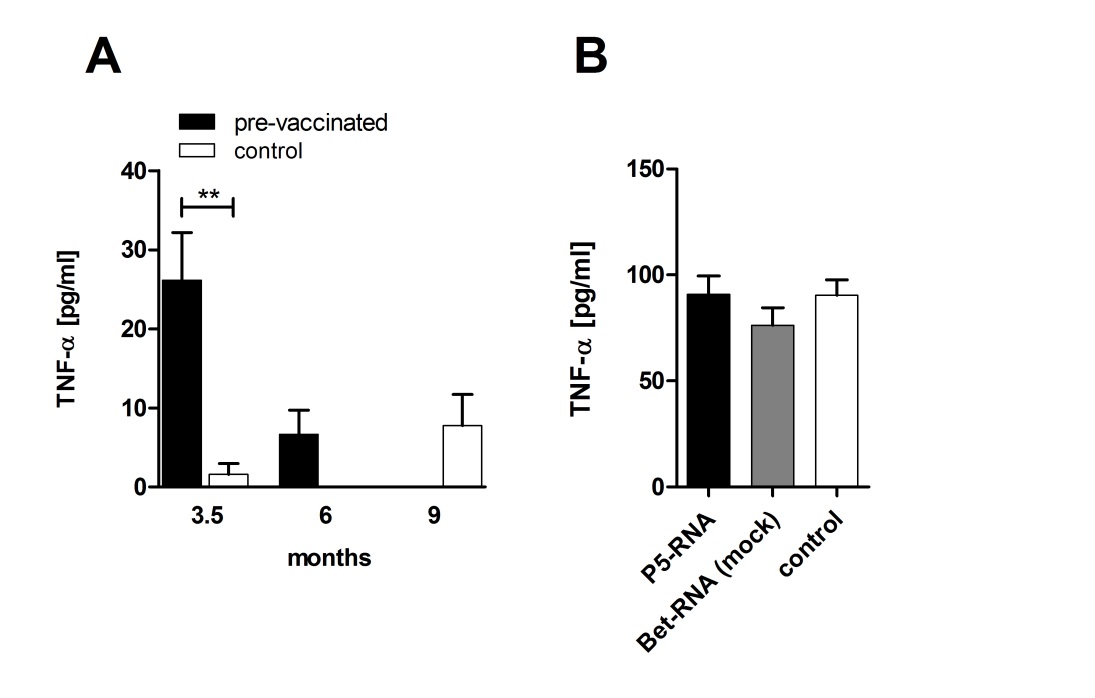


Supplementary Figure S2. TNF-α was determined in culture supernatants after *in vitro* restimulation of splenocytes with Phl p 5. Splenocytes were analyzed after sensitization in mice that had been vaccinated 3.5, 6, or 9 months earlier (A) or after 7 monthly aerosol challenges (B). Data are displayed as means ± SEM (n = 5). ** P < 0.01.
